# Tree rings reveal signs of Europe’s sustainable forest management long before the first historical evidence

**DOI:** 10.1038/s41598-020-78933-8

**Published:** 2020-12-11

**Authors:** Bernhard Muigg, Georgios Skiadaresis, Willy Tegel, Franz Herzig, Paul J. Krusic, Uwe E. Schmidt, Ulf Büntgen

**Affiliations:** 1 Institute of Forest Sciences, Chair of Forest History, Albert-Ludwigs-University Freiburg, Tennenbacher Strasse 4, 79106 Freiburg, Germany; 2 Institute of Forest Sciences, Chair of Sylviculture, Albert-Ludwigs-University Freiburg, 79106 Freiburg, Germany; 3 Institute of Forest Sciences, Chair of Forest Growth, Albert-Ludwigs-University Freiburg, 79106 Freiburg, Germany; 4 Bavarian State Department for Cultural Heritage, 86672 Thierhaupten, Germany; 5 Department of Geography, University of Cambridge, Downing Place, Cambridge, CB2 3EN UK; 6 Swiss Federal Research Institute WSL, Zürcherstr 111, 8903 Birmensdorf, Switzerland; 7 Global Change Research Centre (CzechGlobe), Bělidla 986/4a, 603 00 Brno, Czech Republic; 8 Department of Geography, Faculty of Science, Masaryk University, Kotlářská 2, 613 00 Brno, Czech Republic

**Keywords:** Ecology, Environmental sciences, Environmental social sciences

## Abstract

To satisfy the increasing demand for wood in central Europe during medieval times, a new system of forest management was developed, one far superior to simple coppicing. The adoption of a sophisticated, Coppice-with-Standards (CWS) management practice created a two-storey forest structure that could provide fuelwood as well as construction timber. Here we present a dendrochronological study of actively managed CWS forests in northern Bavaria to detect the radial growth response to cyclical understorey harvesting in overstorey oaks (*Quercus* sp.), so-called *standards*. All modern *standards* exhibit rapid growth releases every circa 30 years, most likely caused by regular understorey management. We further analyse tree-ring width patterns in 2120 oak timbers from historical buildings and archaeological excavations in southern Germany and north-eastern France, dating between 300 and 2015 CE, and succeeded in identifying CWS growth patterns throughout the medieval period. Several potential CWS *standards* even date to the first millennium CE, suggesting CWS management has been in practice long before its first mention in historical documents. Our dendrochronological approach should be expanded routinely to indentify the signature of past forest management practices in archaeological and historical oak wood.

## Introduction

Wood is a key resource for human beings. Aside from its use for the production of tools and various everyday objects, forests have had to satisfy a constant demand for construction timber and fuelwood. Ever since people established a sedentary lifestyle, they were faced with the problem of local wood supply. Consequently, a wide range of sustainable silvicultural systems were developed in different parts of medieval Europe, which can be summarized as “coppice-with-standards” (CWS), cf. “taillis sous futaie” (FR), “Mittelwald” (DE), “monte medio” (ES)^1^.

CWS is a modern expression for an old silvicultural system^2^, which describes a two-storey forest structure combining an understorey of even-aged coppice species harvested in relatively short and regular rotation to provide fuelwood and an uneven-aged upper story of *standard* trees grown at wide spacing for the production of high-quality construction timber^1,3^. The earliest documentary evidence of CWS management in different parts of Germany dates back to the thirteenth century^4–8^.

CWS forest management was widely used in medieval and post-medieval Europe^1,3,9–18^, and remained essential in many parts of the continent until the nineteenth century, when petroleum and natural gas increasingly took over the role of providing the primary fuel source^3^. The decreasing importance of firewood consequently altered the goal of forest management towards timber production^19^. At the beginning of the nineteenth century forestry experts wrote harsh critiques on traditional forest management systems^20–24^. Coppice forests and CWS stands were successively transformed into climax forests with closed canopy until eventually, they had vanished in most regions of Europe by the mid-twentieth century^2,18^. Today there is an increased scientific, economic and political interest in CWS management systems for addressing questions of promoting biodiversity and resource sustainability under climate and environmental change^12,25–29^. Coppice and CWS forests have also been the focus of research in different fields of forest history^16,17^, historical geography^30,31^, anthracology^32,33^, dendrochronology^18,34^, and dendroarchaeology^35,36^. However, dendroarchaeological studies on pre-modern forest management have so far mainly focused on coppice rather than CWS forests^33,36^, and the few dendrochronological studies on CWS management systems were restricted to abandoned CWS stands^18,34^.

**Figure 1:**
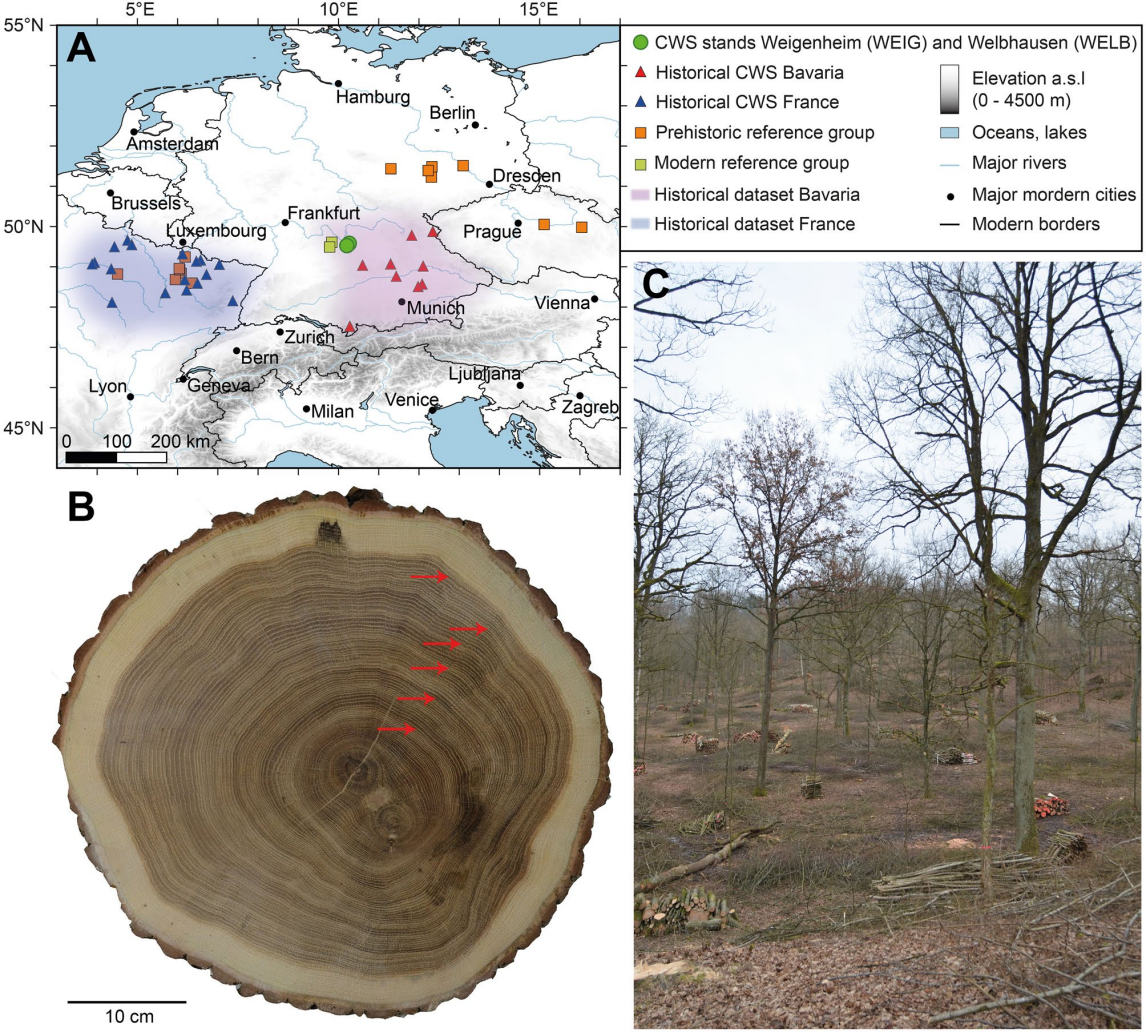
(**A**) Locations of the modern coppice-with-standard (CWS) forest in Weigenheim (WEIG) and Welbhausen (WELB) (green dots), the detected historical CWS standards in Bavaria (red triangles) and north-eastern France (blue triangles), as well as the prehistoric (orange squares) and modern (light green squares) reference groups. For details see supplementary Table S1. The spatial distribution of the historical datasets is indicated by colour shadings in Bavaria (purple) and France (blue). (**B**) Cross-section of an oak standard from Welbhausen (WELB), felled in winter 2017/2018. Visible growth releases are indicated (red arrows) (**C**) CWS forest in Weigenheim (WEIG) during understorey harvest in January 2018. The map was created using QGIS, version 3.4.11-Madeira (https://qgis.org/de/site/).

Here, we analysed 161 oak (*Quercus* sp.) *standards* from Weigenheim (WEIG) and Welbhausen (WELB), located in a small region in northern Bavaria (Fig. 1), one of the few places in central Europe where CWS forests are still maintained by local communities. In this region more than 1500 ha of forest are managed in the traditional CWS system, accounting for more than 4% of the total forest area^37^. This proportion of CWS is unique, because everywhere else in Europe the practice was largely abandoned in the nineteenth century^12^. First, we measure the tree-ring width (TRW) to identify the shape of the growth-release attributable to CWS management and compare it to a randomly sampled collection of oak standard material from two regional sawmills in nearby north-eastern Baden-Württemberg to serve as a modern reference group. We then expand our study to ancient oaks from historical buildings and archaeological excavations in two regions with a long CWS tradition in Bavaria and north-eastern France to detect possible CWS forest structures during medieval times. Finally, we use a prehistoric reference group to verify the skill of our analytical approach.

## Results

**Figure 2:**
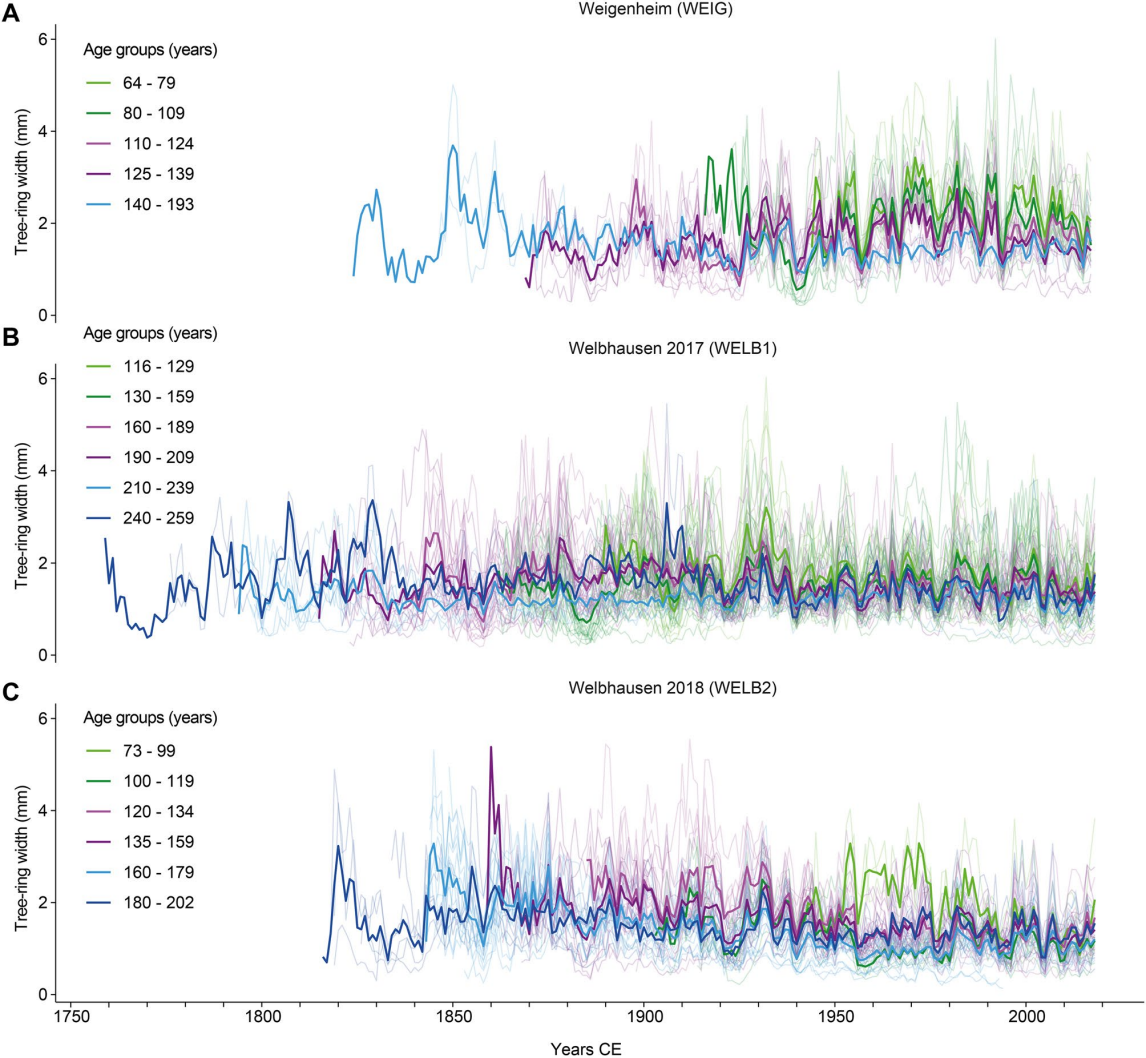
Tree-ring width curves (TRW) from Weigenheim (**A**) and Welbhausen (**B**, **C**) distributed in evenly replicated age groups. Young trees are shown in bright and dark green, middle-aged trees in bright and dark purple and old trees in bright and dark blue. Group means are represented by bold lines, single TRW are displayed in the background (thin semi-transparent lines). This figure was created using R software (https://www.r-project.org/).

From the contemporary CWS dataset, mean TRW chronologies were created for Weigenheim (WEIG) and Welbhausen (WELB1 and WELB2). Due to considerable differences in stand and tree age a division into several evenly replicated age groups was performed (Fig. 2). The individual tree ages of the modern CWS dataset range from 64 to 193 years in Weigenheim (mean 112 years), and from 116 to 259 (mean 167 years) and 73–202 (mean 148 years) in Welbhausen (WELB1 and WELB 2). For further details see Supplementary Table S2. Periodic years of common release events on the stand level are not clearly visible in the mean TRW chronologies (Fig. 2). Hence, further analyses were performed at the tree level.

**Figure 3:**
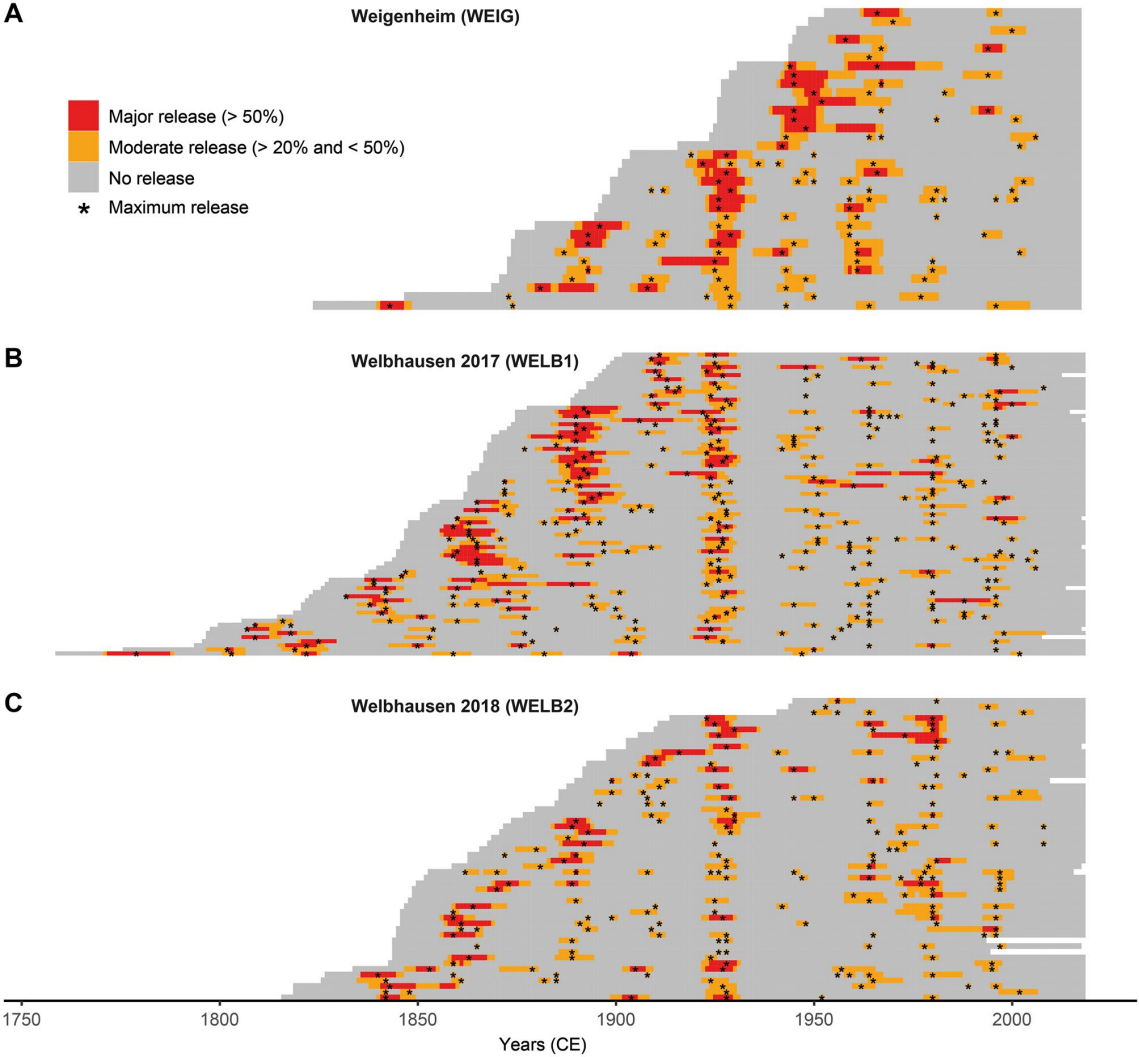
Bar graphs with release events for every tree from Weigenheim (**A**) and Welbhausen (**B**, **C**). Major release events are shown in red, moderate release events in orange. Release maxima for longer release episodes are highlighted (black stars). This figure was created using R software (https://www.r-project.org/).

All 161 samples from the modern CWS dataset show characteristic release events, such as those plainly visible in the stem cross-section shown in Fig. 1B. Applying growth averaging (GA) analysis^38^ (see “Methods”) on the single CWS *standards*, revealed up to nine significant release events (4.23 on average), depending on the individual tree age (Fig. 3). The average interval between release events was 31.2 years, with a standard deviation (SD) of 10.1 years. The intensity of these release events appears to decline with age, with most of the major releases occurring in the early parts of the TRW series (Fig. 3). Notably, there are almost no release events visible in the approximately two past decades (Fig. 3). Based on these results, we refined our CWS detection by scanning the historical TRW data for those samples that contain multiple release events in intervals of 21–41 years (see “Methods”).

**Figure 4:**
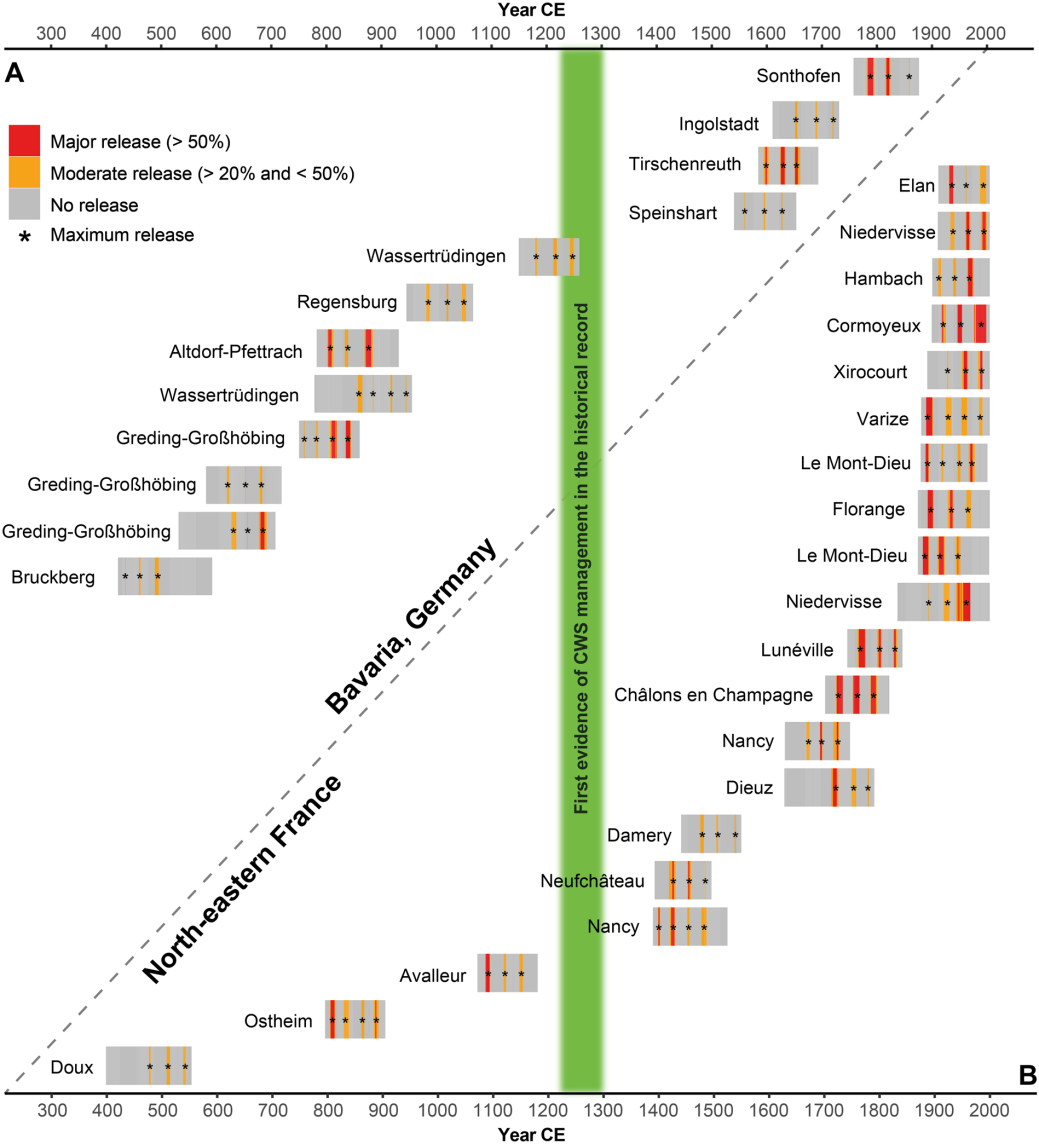
Bar graphs of detected possible CWS standards from the historical dataset for Bavaria (**A**) and north-eastern France (**B**). Major release events are shown in red, moderate release events in orange. Release maxima are highlighted (black stars). The period of early historical records for CWS management is indicated (green). This figure was created using R software (https://www.r-project.org/).

In the historical TRW dataset from Bavaria (995 samples), 12 possible CWS standards (1.2%) were identified. They display an average of 3.2 release events with a mean interval of 30.9 years and a SD of 2.7 years (Fig. 4A). Seven samples (58%) date to the first millennium CE and hence to the period before the first documentary evidence of CWS management. One sample dates to the thirteenth century and four samples date to the period after 1500 CE.

Out of 1125 samples from north-eastern France, 20 (1.8%) have an average of 3.2 release events with an average interval of 30.1 years and a SD of 2.7 years (Fig. 4B). Only three samples date prior to the first documented mention of CWS management (one sample to the sixth century, one to the early tenth century and one to the late twelfth century). Three more samples date to the sixteenth century, four to the modern period eighteenth and nineteenth century and ten samples originate from recent trees.

The same scanning parameters were applied to a modern reference group (Fig. 1A) of 443 regional oak samples from sawmills in Bad Mergentheim (BAME) and Wittighausen (WITT). From this dataset, four individuals (0.9%) passed the scanning criteria with three release events each (Supplementary Fig. S1). The mean interval is 31.1 years with a SD of 2.3 years. Additionally, a prehistoric reference group from the period before 5000 BCE was tested. None of the 115 tree-ring width series from this exceptional collection were found to contain demonstrable, cyclical suppression and release cyles, confirming our hypothesis and indicating that our methodological approach is not susceptible to random release events.

## Discussion

### Sample size and study composition

With 161 modern CWS *standards* and over 2000 historical oak samples, this study provides a rather large dataset compared to other dendroarchaeological studies^36^. CWS management contains an inherent bias towards selection of well-performing, i.e. dominant, trees for *standards*. Therefore, the sampling design may influence the results of dendrochronological studies^39^. However, the historical datasets include only mature trees (i.e. > 100 tree rings) and are therefore presumed to represent a similar selection of dominant trees, via-a-vis a builders preference for large stem diameters to produce suitable construction timbers.

The sampling strategy of cutting discs from close to the crown base was performed to avoid reducing the merchantable value of the remaining stems (as opposed to breast height, i.e. 1.3 m above ground). Therefore the heights at which each modern stem disc is collected differs considerably from the height commonly used in forest sciences and dendroecology^40^. It has been demonstrated that the choice of a different sampling height has little influence on the TRW ratio^33^. However, we need to stress that when sampling a tree anywhere above ground level, the number of tree rings from pith to bark does not reflect the actual tree age. Nevertheless, a rough grouping of *standard* generations is noticeable both in WEIG and WELB1 (Fig. 3A,B). The modern reference group consists of 443 samples collected from regional sawmills (Fig. 1A). CWS detection was confirmed in 0.9% of the samples (Supplementary Fig. S1), showing that the inclusion of samples from CWS forests cannot be ruled out. The dataset provides no information on the stand conditions, making it an ideal reference group for our study. The lack of stand information and provenance is symptomatic of samples from historical buildings and archaeological features^41^. Therefore, any attempts to compare release effects need to be made on the individual tree level instead of stand level^18,34^. The “random sampling” approach applied on the modern oaks ensures a comparable quality of tree-level information with the historical and archaeological oak samples^42^.

The historical datasets cover the period 300–2015 CE and are equally well replicated with 995 samples from Bavaria and 1125 samples from France. Significant positive correlations between the historical datasets (r = 0.42, p > 0.001) substantiate the comparability between both regions. The prehistoric reference dataset comprises 115 samples from central Europe (Fig. 1A).

### Differences and uncertainties in rotation cycles

The detected cycle length of 31.2 years on average for the CWS dataset corresponds with the documented cycle lengths of 28 years (WEIG) and 34 years (WELB)^43^. However, considerable differences exist regarding the timing of release events among trees on the stand level (Figs. 2 and 3). Contrary to the theoretical model of CWS management, forest stands might have experienced diverse variations in rotation cycles according to regional variations in local demands and external factors, which is reflected in a high average SD of 10.1 years. One example of the variation in cycle lengths is found in the WELB1 collection, where *standards* scheduled for felling in winter 2017/2018 remained standing until autumn 2018 due to unfavourable weather conditions.

Given these uncertainties and lacking explicit archival data on CWS understorey fellings for earlier management cycles, we aimed for a tree-level approach. This enabled the assessment of historical and archaeological timber and allowed the detection of individual trees which most likely originated from CWS forests. Based on the results obtained from modern CWS stands (with a mean interval of 31.2 years between consecutive release events and SD of 10.1 years), we screened the historical datasets for samples exhibiting more than two consecutive release events occurring at an average interval of 26–36 years (SD < 5). This approach would detect a hypothetical TRW series with four release events: the second occurring 32 years following the first; the third occurring 25 years following the second; and the fourth occurring 30 years after the third (mean interval of 29 years between the release events and SD of 3.6 years). Applying these criteria, we expected to detect all rotation cycles between 21 and 41 years. Nevertheless, the GA method does contain an inherent lack of sensitivity in detecting short rotation cycles less than 10 years^34,38^. The length of historically documented understorey felling cycles varies greatly between five and 40 years, depending on region and period^17,25^. Some authors have mentioned a tendency for shorter rotation cycles in medieval times^1,18,44^. It is important to note that these shorter cycles cannot possibly be detected with the method applied here.

Furthermore, there are explicit historically recorded examples of variable understorey felling cycles within the same forest^17^. The length of rotation periods might have been adjusted in response to varying demands for wood, the productivity of a given stand but also considering the sustainable provision of wood. Additionally, there are a variety of historically recorded uses of forest by-products with possible effects on tree growth, from hog feeding^45^, grazing and pollarding^46^ to litter raking that might have culminated in soil degradation^47^. In the forests of Weigenheim and Welbhausen, silvopastoral use traditionally did not play a significant role^37^. The practice of litter raking in the Welbhausen CWS stand is historically recorded in 1601 CE^43^ and thus long before the growing period of the studied trees. Our study aimed to detect signals of CWS management in modern and relic TRW series. Further studies on explicit stands, however, must take the use of by-products into account.

### CWS signal distinction and release event detection

This study comprises the first attempt to identify and extract a CWS signal from oak *standards* growing in actively managed CWS forests, providing an improved understanding of their annual stem-growth patterns. The observed absence of release effects in the studied CWS *standards* for several years prior to their felling (Fig. 3) provides strong indications that the release effects recorded in the TRW sequences are in fact associated with understorey felling activities^18^. Several other biotic, such as insect infestations^48^, or abiotic factors, including windfalls after storms^49^ and hydroclimatic extremes^50,51^ might cause growth releases similar to the ones observed in CWS *standards*. Periodic insect infestations can be ruled out due to shorter interval lengths^52,53^. Regarding wind throw, we compared the number of release events per year with recorded storms affecting southern Germany since 1800 but found no discernible relationship (Supplementary Fig. S2). Climatic factors might have influenced the frequency of release events for example in the period 1920–1930 visible in many of the CWS trees (Fig. 3). However, during this period the observed release maxima are spread over a decade and therefore unlikely related to annual reactions to environmental conditions or (hydro)climatic events that in theory should affect all trees within a wider area almost simultaneously^54^.

There are some periods with less distinct or concurrent release events in the second half of the 19th and the first decades of the twentieth century. These more equivocal examples are mostly visible in the oldest individuals from the Welbhausen datasets (Fig. 3B,C) and possibly associated with (1) restructuring in the local CWS practices in the 1920s^43^, (2) when the felling cycles were probably disturbed by World War II in the 1940s, and (3) during the last approximately 50 years. One possible explanation for the decreasing response of *standards* to understorey harvesting within the last two rotation cycles (Fig. 3) is the elaborate root and crown systems of the artificially dominant oaks that might be affected less by the understorey vegetation after reaching a certain age and height^38^. Such a decreasing growth response to coppice harvesting was clearly visible when *standards* are grouped into age classes for each stand (Fig. 2). Another reason for the decrease in response might also be the decline of inter-cyclic maintenance measures (e.g. coppice thinning within a rotation cycle) in the past half century^43^.

The second element determining a CWS-signal is the periodical occurrence of release events. The CWS *standards* analysed show up to nine release events over a period of up to 259 years, occurring every 31.2 years on average (SD 10.1 years) and showing a clear cyclical pattern (Fig. 3). However, the coordinated release events of single trees, spread over several years, illustrate how variable the individual growth reaction of trees within the same forest stand can be (Fig. 3), thereby supporting our single tree approach for CWS detection in the historical datasets.

To identify CWS *standards* in historical wood samples without stand information, we applied strict scanning criteria. The requirement that all samples have 100 or more tree rings aims to preferentially select mature oaks with potential to capturing three or more harvest cycles: To determine periodical releases and an average duration of the management cycle, a minimum of three consecutive release events had to be present in one sample.

The results from the historical datasets indicate CWS management was practised throughout the medieval period in both north-eastern France and Bavaria, even though the explicit proofs are sporadic and isolated. Both areas show differences in the chronological distribution of the detected CWS occurrences. The Bavarian dataset provides more evidence for early medieval CWS *standards*. Three of the Bavarian samples from the first millennium originate from excavations in Greding-Großhöbing, an area continuously exploited for timber and iron production during the early Middle Ages^55,56^. Half the identified CWS *standards* from France originate from recently felled trees, reflecting the bigger role of traditional oak forests in modern France^57^. Consequently, these results are not considered as representative of the actual prevalence of CWS forest structures in medieval central Europe^9,16^. The total amount of identified CWS *standards* is conspicuously low (1.2% and 1.8% for Bavaria and France, respectively) but greater than what would be expected by chance alone (the probability of detecting a cyclical growth release pattern in the historical dataset by chance = 0.7%). In comparison, the modern reference group shows a 0.9% probability of CWS *standard detection*. This suggests that the scanning criteria applied may be too strict. However, to avoid possible overinterpretation of casual release effects, we kept the criteria obtained from the modern CWS *standards*. The prehistoric reference group consists of relic and archaeological samples dating to the 8th–6th millennium BCE and presumably represents trees from primary forests. The first sedentary societies in Europe started exploiting these forests during the second half of the 6th millenniums^58^. The prehistoric reference group was therefore expected not to reflect any signs of forest management, which was verified by our results.

Management-induced growth patterns might furthermore influence the climatic signal within historical oak samples^59,60^. It is therefore crucial to overcome these patterns in paleoclimate reconstructions with sufficient sample replication^61^. Our results find negligibly low proportions of CWS managed oaks in the historical datasets. The few examples, however, demonstrate that CWS management needs to be considered when studying historical forest management for the Middle Ages. Further studies on historical CWS management may, therefore, address the question of suitability for climate reconstructions.

### Indications for CWS systems before the historical record

The first historical mentions of CWS management in written sources date to the thirteenth century CE^6,8^. Early empirical evidence of simple coppice management systems date to prehistoric times, at least from Late Bronze age onwards^62^. Written sources from Roman times describe coppice forests (*silvae caeduae*)^63^ and etymological indications suggest coppice use in the Early Medieval period^19,64^. It is striking that the first historical evidence describing simple coppice forests in medieval central Europe date from the thirteenth century, e.g. in the cities of Aachen (1215 CE), Speyer (1219 CE) and Erfurt (1264 CE)^8,19^, indicating that accounts detailing forest management in general were not the subject matter of documentary sources prior to the High Middle Ages (approximately 1000–1250 CE). Therefore, dendroarchaeological evidence provides the only explicit sources for historical forest management in earlier periods. Given our new results, a reinterpretation of written sources might provide further indications of early CWS management. For example, a document from Bichel near Salzburg, Austria, written in 923 CE, distinguishes between *silvae* (forests) and *arbustae* (shrublands) and thus indicates a two-storey forest structure^6,43^.

Bernard^13^ addresses the period from the sixth to ninth century as an era of “proto-silviculture”, based on the observation of massive fellings over several centuries in France without a noticeable decline in timber quality. Until now, in Germany, there were only indirect references of CWS-like management practises mentioned by several authors around 600 CE in the context of hog feeding^1,9,29^. A distinction between *ligna fructifera* (fruit-bearing trees) and *ligna infructifera* (non-fruit-bearing trees) is made in the *leges,* germanic tribal laws dating between fifth and ninth century CE^43,65,66^. To produce acorns (for generative reproduction as well as for example pig masting) oaks need to reach an age of 40–80 years, and thrive in an optimal stocking level^67^. Hence, CWS-like forest structure with its typical wide spacing might have favoured acorn-production and, therefore, hog feeding. The dual use for wood and masting, however, requires to some extent the protection of acorn-bearing trees. For this reason, Hausrath^6^ considers pasture forests as the preliminary stage for the development of CWS forest structures.

Woodlands in close vicinity of settlements were regularly and continuously used for both wood exploitation and forest pasture^68,69^. Ninth century documents define the size of a forest by the number of pigs fed on its mast crop, reflecting the high importance of forest pasture during the early medieval period^8,66^. In combination with the constant demand of fuelwood from forests in close vicinity to settlements, this possibly indicates a two-storey forest structure with two seperate harvesting cycles, which defines a CWS forest. Yet, the division of a forest area into fixed annual cuts, characteristic for modern CWS forests, cannot be evinced before the fifteenth century^8^. However, given the combined use for pasture and wood production, certain short-term or permanent installations (e.g. cattle fencing) may have modulated annual cutting areas^44^.

## Conclusions and outlook

This study, for the first time, shows the potential of TRW-studies to detect historical CWS management systems. The growth patterns extracted from currently managed CWS *standards* allow a better understanding of growth reactions in CWS forests. Such a tree-level approach enables future studies of archaeologically excavated construction timber with regard to silvicultural practices. Therefore, this study provides important new information on the resource management, the organisation of rural subsistence societies and landscape history. Last but not least, our dendrochronological approach should be expanded routinely to identify the signature of past forest management practices in archaeological and historical oak wood.

## Methods

### Regional settings and datasets

The study area for our recent CWS dataset is located close to the town of Uffenheim in northern Bavaria (Fig. 1). The dataset consists of 161 oak samples from two forests in Weigenheim (WEIG) and Welbhausen (WELB) (Fig. 1A). Each forest is managed by a different constellation of cooperative organisation, similar to the management systems of medieval community forests^17,37^. The measuring and sizing of cutting areas is conducted by a defined number of partially elected co-owners with the help of a wooden measurement rod of a specified length. Fairness in the distribution of fuel wood is provided by drawing lots and different forms of compensation^43^.

The CWS forest of Weigenheim covers a total of 220 ha and is managed by a prescribed rotation cycle of 28 years. We collected stem discs from 36 *standards* harvested in winter 2017/2018 (WEIG). The municipal forest of Welbhausen, an area of 170 ha is also managed under a CWS system with a rotation cycle of approximately 34 years. The earliest written sources of CWS in this forest dates back to 1447 CE, with the most recent regulation written in 1929^43^. From this forest we collected the larger part of our samples from two adjacent cutting areas (WELB1, 73 samples and WELB2, 52 samples).

TRW series from archaeological and historical timbers from the period 300–2015 CE originate from north-eastern France and Bavaria (Fig. 1A). The historical dataset is comprised of mature oaks (100 tree rings or more) from various archaeological structures and historical buildings from the Late Roman Period to modern times collected within the last decades^42,70^. The datasets, consisting of 995 samples from Bavaria and 1125 samples from north-eastern France, allow for a multi-period survey of possible CWS management practices. The modern reference group consists of 443 oak samples from sawmills in the region (Fig. 1A). The prehistoric reference group originates from several sites in central Europe with archaeological and subfossil samples dating to the 8th to 6th millennium BCE (Fig. 1A). This last group consists of 115 oak samples that are expected to show no CWS signal.

### Sample collection and TRW measurement

Sample collection in the CWS stands (WEIG, WELB1 and WELB2) was performed on freshly felled trees by cutting stem discs close to lower crown height following the “random sampling” approach^42^. All samples were prepared following standard dendrochronological methods^71^. TRW was measured with a precision of 0.01 mm using binocular microscopes and a semi-automatic measuring table. The TRW series were crossdated using the PAST software^72^.

### Release event detection

Understorey coppicing in a CWS forest is associated with increased growth (commonly referred to as release) in the remaining trees (*standards*) due to reduced competition for light, nutrients and water^18^. To detect such positive growth reactions, we adapted the growth averaging (GA) method developed by Nowacki and Abrams^38^. Our approach focused on single tree reactions rather than the stand level, which enables a comparison with archaeological material. We performed running comparisons of consecutive 10-year TRW means. These were used to detect sustained growth increases following canopy openings as opposed to abrupt growth changes caused by other factors, such as climatic extremes^38^. For each year in each TRW series, percentage of growth change (%GC) was calculated using the equation proposed by Nowacki and Abrams^38^:

$$\% {\text{GC}} = \left[ {{{\left( {M_{2} - M_{1} } \right)} \mathord{\left/ {\vphantom {{\left( {M_{2} - M_{1} } \right)} {M_{1} }}} \right. \kern-\nulldelimiterspace} {M_{1} }}} \right] \times {1}00$$

where %GC is the percentage of growth change between preceeding and subsequent 10-year means, *M*_*1*_ preceding 10-year mean including the target year and *M*_*2*_ subsequent 10-year mean. Following this approach, the periods for instance *M*_*1*_ = 1991–2000 and *M*_*2*_ = 2001–2010 would be used to calcucate %GC at the year 2000.

Similar to^18,34,38^, major release events were considered to be years with %GC greater than 50%. Years when %GC exceeded 20% were considered moderate releases. In cases when multiple consecutive years were detected as release events, we considered the year with maximum %GC (release maximum) as the release date.

To detect signals of CWS in the individual historical and archaeological samples, we first detected growth releases in the individual TRW series by applying the adapted GA method, as described above for the modern CWS dataset. Since management practices for the forests where historical and archaeological samples originated from are unknown, we used the results obtained from the modern CWS TRW series to detect patterns in the occurrence of release events that could indicate CWS practices. Therefore, we identified the individual series in the historical dataset with three or more consecutive release events, occurring at an average chronological interval of 26–36 years with less than 5 years standard deviation (SD). Only tree-ring series with three or more release events were considered to distinguish cyclic management signals from other release events.

## Supplementary Information

Supplementary Information.
