# Supplementary material for: Tree rings reveal signs of Europe’s sustainable forest management long before the first historical evidence
Source: Sci Rep. 2020 Dec 11;10:21832. doi: 10.1038/s41598-020-78933-8 (PMC7733517; doi:10.1038/s41598-020-78933-8)
Supplement: Supplementary file 1 — Supplementary Information. [file 41598_2020_78933_MOESM1_ESM.pdf]

Supplementary information for:

Tree rings reveal signs of Europe's sustainable forest management long before the first historical evidence

Bernhard Muigg, Georgios Skiadaresis, Willy Tegel, Franz Herzig, Paul J. Krusic, Uwe E. Schmidt, Ulf Büntgen

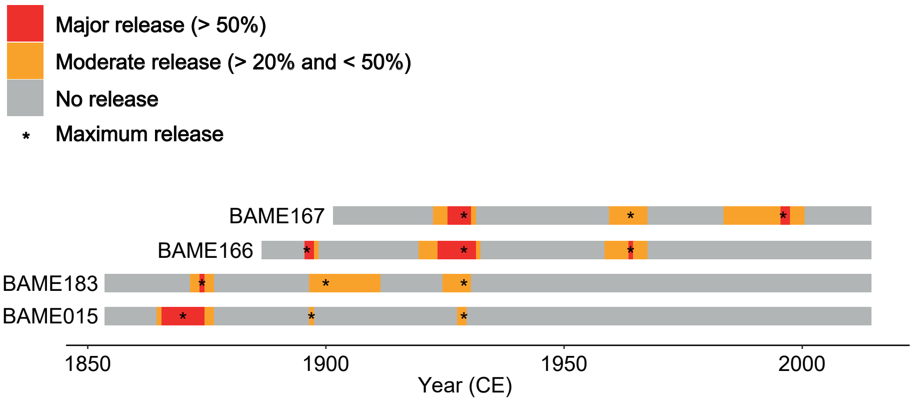

Supplementary Fig. S1: Detected CWS standards from the modern reference group. From 443 samples collected from two sawmills in Bad Mergentheim (BAME) and Wittighausen (WITT), a total of four tree individuals (0.9%), all from BAME, were found showing CWS growth pattern. No CWS standards were detected from WITT. This figure was created using R software (https://www.r-project.org/).

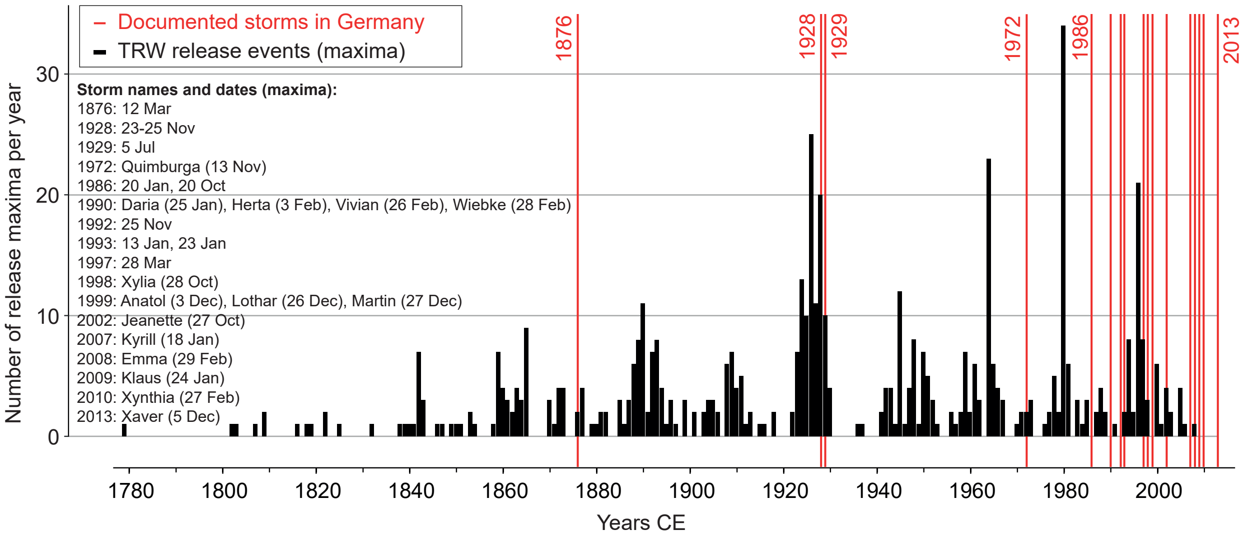

Supplementary Fig. S2: Synchronisation of detected release event maxima from tree-ring widths (this study) and heavy storms that affected Germany since 1800 CE. Storm data since 1981 from: http://www.europeanwindstorms.org/ (retrieved 24.09.2020) For earlier events see: Kienitz 1877, Berhardt 1880, Cappel and Emmerich 1975, cf. Donat et al 2010.

Bernhardt A (1880) Waldbeschädigungen durch Sturm- und Schneebruch in den deutschen Forsten während der zehn Jahre 1868 - 1877. Suppl. zur Allg. Forst- und Jagdzeitung XI, 53 - 142.
Cappel A and Emmerich P (1975) Zwei Wetterkatastrophen des Jahres 1972: der Niedersachsen-Orkan und das Gewitterunwetter von Stuttgart. Rep German Weather Serv 135, Offenbach
Donat MG, Leckebusch GC, Wild S, Ulbirch U (2010) Benefits and limitations of regional multi-model ensembles for storm loss estimations. Climate Research 44, 211-225. DOI: 10.3354/cr00891
Kienitz M (1877) Beobachtungen über den Sturm vom 12. März 1876 und den durch denselben in der Oberförsterei Marburg in Hessen verursachten Schaden. Allg. Forst- und Jagdzeitung, 365.

Supplementary Table S1: Sites and coordinates of detected historical CWS from France (blue) and Bavaria (red) indicated by traingles in Fig. 1A. For chronological information see Fig. 4.
Below: sites and coordinates of prehistoric (orange) and modern (green) reference data, indicated by rectangles in Fig. 1A.

	Site/Location	N	E
Historical CWS in France	Niedervisse-Scierie	49,168017	6,57104
	Niedervisse-Scierie	49,168017	6,57104
	Hambach-Scierie chêne de l'est	49,061192	7,037424
	Varize-Scierie Laglasse	49,139721	6,466875
	Elan-Scierie Potier Père et Fils	49,66	4,755
	Xirocourt-Mittelwald	48,4234	6,2228
	Florange-Forêt	49,32	6,122
	Cormoyeux-Forêt Talma	49,110185	3,93664
	Le Mont-Dieu-Forêt Dom	49,55	4,86
	Lunéville, Château Ducal	48.594	6.492
	Châlons en Champagne-9-11rue Herbillon - 4 rue Lochet	48,95	4,35
	Dieuz-Les Salines Royales	48,812	6,717
	Nancy-Pont d´Essay	48,68	6,17
	Damery-St.Georges	49,071	3,876
	Nancy-Couvent des Cordeliers	48,68	6,18
	Neufchâteau-45 Rue Saint Jean	48,354699	5,69399
	Chapelle d'Avalleur	48,12	4,37
	Ostheim-Birgelsgaerten	48,159	7,37
	Doux-La Cayabre	49,5	4,43
Historical CWS in Bavaria	Sonthofen, Burgberger Brücke	47,524041	10,281232
	Ingolstadt, Gießereigelände	48,765657	11,433579
	Tirschenreuth, Sanierung Fischhofbrücke	49,880027	12,342459
	Wassertrüdingen, Neubau Altstadtzentrum	49,040396	10,59764
	Speinshart, Kloster	49,785974	11,820173
	Wassertrüdingen, Wörnitzaue BB2011	49,039859	10,597708
	Altdorf-Pfettrach	48,572628	12,078667
	Regensburg, Museum der Bayerischen Geschichte	49,020195	12,102468
	Greding-Großhöbing, Flurstelle 74	49,072613	11,294763
	Greding-Großhöbing, Flurstelle 255	49,072114	11,296008
	Bruckberg-Edlkofen, Kiesgrube	48,511729	11,978478
Prehistoric reference group	Dieulouard-Sablière, FR	48,840434	6,086889
	Gondreville, FR	48,684817	5,944145
	Norroy-lès-Pont-à-Mousson, FR	48,929535	6,052004
	Rosières-aux-Salines, FR	48,593931	6,347404
	St. Martin-aux-Champ, FR	48,82189	4,507314
	Vandières, FR	48,957787	6,041115
	Ay sur Moselle-Sablière, FR	49,245237	6,185119
	Altscherbitz, DE	51,393114	12,228148
	Elbekilometer 153,325, DE	51,513377	13,096038
	Oberröblingen-Sangerhausen, DE	51,435755	11,300882
	Zwenkau-Eythra, DE	51,228722	12,306053
	Brodau, DE	51,486547	12,320931
	Ostrov, CZ	49,981411	16,04617
	Unicov, CZ	49,76583	17,095387
	Velim, CZ	50,054125	15,108789
Modern reference group	Wittighausen (WITT), DE	49,610	9,832
	Bad Mergentheim (BAME), DE	49,478	9,772

Supplementary Table S2: Chronology statistics and characteristics for all datasets: SL= segment length; SD = standard deviation; AGR = average growth rate (TRW); mean IC = mean inter-series correlation; n(trees) = total number of trees; EPS = expressed population signal; SNR = signal-to-noise ratio. Line 1-3: Modern Coppice-with-standards (CWS) datasets (green). Line 4-5: Historical datasets from France (light blue) and Bavaria (light purple). Line 6-7: Prehistoric (orange) and modern (light green) reference groups (Colours are the same as in Fig. 1A)

	Start year	End year	mean SL(SD)	AGR(SD)	mean IC	n(trees)	rbar	EPS	SNR
**CWS Weigenheim (WEIG)**	1824	2017	112.4(31.7)	1.80(0.78)	0.65	34	0.36	0.92	11.01
**CWS Welbhausen 2017 (WELB1)**	1759	2018	166.8(31.9)	1.56(0.69)	0.52	74	0.22	0.93	13.29
**CWS Welbhausen 2018 (WELB2)**	1816	2018	148.3(31.4)	1.51(0.70)	0.58	53	0.36	0.96	21.85
**Historical France**	306	2017	139.8(34.3)	1.31(0.77)	0.43	1125	0.22	0.96	25.60
**Historical Bavaria**	307	2015	158.5(52.4)	1.12(0.49)	0.49	995	0.23	0.96	26.87
**Prehistoric reference group**	-7869	-5089	160.3(40.3)	1.44(0.75)	0.43	115	0.30	0.74	2.88
**Mod. ref. Wittighausen (WITT)**	1769	2014	133.2(33.8)	1.38(0.68)	0.56	233	0.30	0.98	54.18
**Mod. ref. Bad Mergentheim (BAME)**	1731	2014	147.1(35.6)	1.28(0.63)	0.56	210	0.30	0.98	46.59
